# Structure of Human DNA Polymerase κ Inserting dATP Opposite an 8-OxoG DNA Lesion

**DOI:** 10.1371/journal.pone.0005766

**Published:** 2009-06-02

**Authors:** Rodrigo Vasquez-Del Carpio, Timothy D. Silverstein, Samer Lone, Michael K. Swan, Jayati R. Choudhury, Robert E. Johnson, Satya Prakash, Louise Prakash, Aneel K. Aggarwal

**Affiliations:** 1 Department of Structural & Chemical Biology, Mount Sinai School of Medicine, New York, New York, United States of America; 2 Department of Biochemistry and Molecular Biology, University of Texas Medical Branch, Galveston, Texas, United States of America; Swiss Federal Institute of Technology Lausanne, Switzerland

## Abstract

**Background:**

Oxygen-free radicals formed during normal aerobic cellular metabolism attack bases in DNA and 7,8-dihydro-8-oxoguanine (8-oxoG) is one of the major lesions formed. It is amongst the most mutagenic lesions in cells because of its dual coding potential, wherein 8-oxoG(*syn*) can pair with an A in addition to normal base pairing of 8-oxoG(*anti*) with a C. Human DNA polymerase κ (Polκ) is a member of the newly discovered Y-family of DNA polymerases that possess the ability to replicate through DNA lesions. To understand the basis of Polκ's preference for insertion of an A opposite 8-oxoG lesion, we have solved the structure of Polκ in ternary complex with a template-primer presenting 8-oxoG in the active site and with dATP as the incoming nucleotide.

**Methodology and Principal Findings:**

We show that the Polκ active site is well-adapted to accommodate 8-oxoG in the *syn* conformation. That is, the polymerase and the bound template-primer are almost identical in their conformations to that in the ternary complex with undamaged DNA. There is no steric hindrance to accommodating 8-oxoG in the *syn* conformation for Hoogsteen base-paring with incoming dATP.

**Conclusions and Significance:**

The structure we present here is the first for a eukaryotic translesion synthesis (TLS) DNA polymerase with an 8-oxoG:A base pair in the active site. The structure shows why Polκ is more efficient at inserting an A opposite the 8-oxoG lesion than a C. The structure also provides a basis for why Polκ is more efficient at inserting an A opposite the lesion than other Y-family DNA polymerases.

## Introduction

Oxidative damage to DNA has been proposed to have a role in cancer and ageing [1]. Oxygen-free radicals formed during normal aerobic cellular metabolism attack bases in DNA and 7,8-dihydro-8-oxoguanine (8-oxoG) is one of the most common adducts formed [2], [3]. Although the high-fidelity replicative DNA polymerases (Pols) can insert an A opposite 8-oxoG, they are inhibited very considerably at both the nucleotide insertion and subsequent extension steps. The recently discovered Y-family of DNA polymerases permit the continuity of the replication fork by allowing replication through such lesions that impede the replicative polymerases [4]. Humans have four Y-family polymerases – Polη, Polι, Polκ, and Rev1 – each with a unique DNA damage bypass and fidelity profile. Polη, for example, is unique in its ability to replicate through an ultraviolet (UV)-induced *cis-syn* thymine-thymine (T-T) dimer by inserting two As opposite the two Ts of the dimer with the same efficiency and accuracy as opposite undamaged Ts [5]–[8]. Because of the involvement of Polη in promoting error-free replication through cyclobutane pyrimidine dimers, its inactivation in humans causes the variant form of xeroderma pigmentosum, a genetic disorder characterized by a greatly enhanced predisposition to sun induced skin cancers [9], [10]. Polι, on the other hand, is unable to replicate through a *cis-syn* T-T dimer but it can proficiently incorporate nucleotides opposite N^2^-adducted guanines and opposite adducts such as 1, N6-ethanodeoxyadenosine which impair the ability of the purine to engage in Watson-Crick (W-C) base-pairing [11]–[15]. Rev1 is highly specialized for incorporation of C opposite template G and promotes efficient dCTP incorporation opposite bulky N^2^-dG adducts via a protein-template directed mechanism of DNA synthesis [16]–[19]. In all, Y-family polymerases in eukaryotes display a large degree of functional divergence, rendering them highly specialized for specific roles in lesion bypass [4].

Polκ is the only human Y-family polymerase with homologues in prokaryotes and archaea, including DinB (PolIV) in *Escherichia coli* and Dbh and Dpo4 in *Sufolobus solfataricus* [20]–[22]. However, the amino acid (aa) sequence of Polκ differs from PolIV and Dpo4 (and other Y-family polymerases) by an extension at the N-terminus of approximately 75 amino acids [23]. This N-terminal extension is indispensable for Polκ activity and is conserved only amongst eukaryotic Polκ proteins. The crystal structure of Polκ in ternary complex with a template-primer DNA and an incoming nucleotide, reveals encirclement of the DNA by this unique N-terminal extension, referred to as the N-clasp [24]. The N-clasp effectively locks the polymerase around the template-primer, perhaps as a means to keep it engaged on a sugar-phosphate backbone distorted by a DNA lesion.

Biochemical studies with yeast and human Y family polymerases indicate that Polη and Polκ have the most proficient ability to replicate through the 8-oxoG lesion [4]. However, whereas yeast and human Polη replicate through 8-oxoG by predominantly inserting a C [25], human Polκ is more efficient at inserting an A opposite the lesion than a C [26]. In this respect, Polκ differs even from Dpo4 (its homologue in *Sufolobus solfataricus*) which prefers to insert a C opposite 8-oxoG [27], [28]. To understand the basis of Polκ's preference for insertion of A opposite 8-oxoG, we have solved the structure of Polκ in ternary complex with a template-primer presenting 8-oxoG in the active site and with dATP as the incoming nucleotide. We show that the Polκ active site is well-adapted to accommodate the 8-oxoG lesion in the *syn* conformation for base pairing with incoming dATP.

## Results

### Structure determination

We crystallized the Polκ catalytic core (aa 19-526) in ternary complex with a 13-nt/18-nt primer/template presenting the 8-oxoG lesion as the templating base and with dATP as the incoming nucleotide. The cocrystals diffract to 3.2 Å resolution with synchrotron radiation (Brookhaven National Laboratory) and there are two ternary complexes (A and B) in the crystallographic asymmetric unit (Table 1). The structure was determined by molecular replacement using the polymerase from the Polκ ternary complex with undamaged DNA as a search model [24]. Electron density maps showed clear densities for the bound DNA, incoming dATP, and the 8-oxoG lesion. For ternary complex A, the final model consists of residues 25–224 and 281–518 of Polκ, nucleotides 2–18 of the template, nucleotides 1–13 of the primer, incoming dATP, and 2 Mg^2+^ ions. For ternary complex B, the final model consists of residues 22–223 and 282–519 of Polκ, nucleotides 2–17 of the template, nucleotides 2–13 of the primer, incoming dATP, and 2 Mg^2+^ ions. The two complexes in the asymmetric unit are similar in structure, though complex A is better ordered and complex B is more complete. We describe below the structure of complex A and refer to complex B as needed.

**Table 1:** 

**Data Collection**		
Wavelength (Å)	1.100	
Resolution (Å)	3.2	
Number of measured reflections	263,685	
Number of unique reflections	34,388	
Data coverage (%)^a^	100 (100)	
R_sym_ (%)^b^	12.1 (46.0)	
I/σ	26.8 (7.4)	
**Refinement Statistics**		
Resolution range (Å)	50.0–3.2	
Reflections	32,684	
R_cryst_ (%)^c^	22.9	
R_free_ (%)^d^	28.6	
Nonhydrogen atoms	Mol A	Mol B
Protein	3,506	3,498
DNA	527	529
dATP	30	30
Mg^2+^	2	2
Water	11	7
Rms Deviations		
Bonds (Å)	0.0118	
Angles (°)	1.52	
Ramachandran plot quality	Mol A	Mol B
Most favored (%)	85.8	82.9
Additional allowed (%)	12.5	15.1
Generously allowed (%)	1.5	1.7
Disallowed (%)	0.2	0.2

a Values for outermost shells are given in parentheses.

b R_sym_ = Σ|I−<I>|/ΣI, where I is the integrated intensity of a given intensity.

c R_cryst_ = Σ||F_observed_|−|F_calculated_||/Σ|F_observed_|.

d R_free_ was calculated using 5% random data omitted from the refinement.

### Overall arrangement

**Figure 1:**
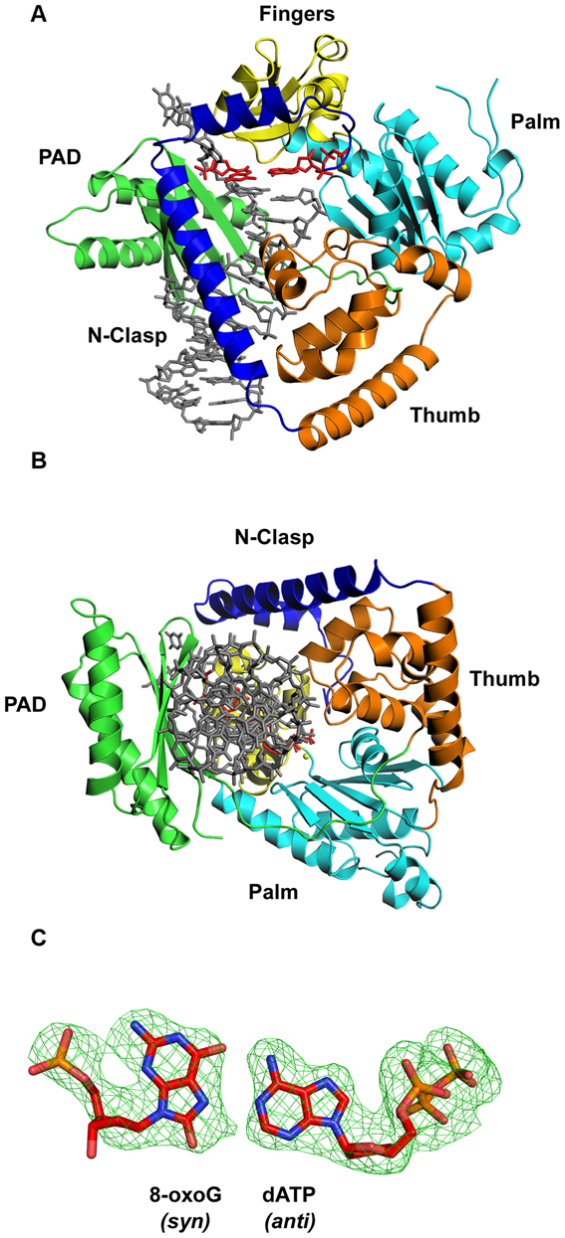
Polκ/8-oxoG/dATP ternary complex. (A) Ribbon diagram representing the overall structure of the ternary complex. The palm, fingers, thumb, PAD and N-clasp domains are shown in cyan, yellow, orange, green, and blue, respectively. DNA is shown in gray and the 8-oxoG lesion and incoming dATP are shown in red. A putative Mg^2+^ ion is shown as a yellow sphere. (B) A view of the ternary complex looking down the DNA helix to show encirclement of the adducted DNA by the N-clasp. (C) Simulated annealing Fo-Fc omit map (contoured at 3.5σ) of 8-oxoG and incoming dATP.

Polκ encircles the 8-oxoG adducted DNA in much the same way as in the ternary complex with undamaged DNA [24]. That is, the conventional right-handed grip on the template-primer by the palm, fingers, and thumb domains, and the PAD (polymerase associated domain), is augmented by an N-clasp subdomain (aa 25–74) that extends from the thumb domain and traverses across the template-primer to the PAD side of the DNA (Fig. 1). The palm and fingers domains interact primarily with the replicative end of the template-primer, wherein the palm (aa 101–109 and 171–338) carries the active site residues (Asp107, Asp198 and Glu199) that catalyze the nucleotidyl transfer reaction, and the fingers domain (aa 110–170) lies over the nascent base pair in the active site formed between 8-oxoG and incoming dATP (described below). The thumb and the PAD straddle the duplex portion of the template-primer, connected by a long linker that cradles one side of the DNA. The thumb (aa 79–100 and 339–401) skims the minor groove surface, while the PAD (aa 401–518) anchors in the major groove (Fig. 1). The majority of DNA interactions are mediated by the PAD, wherein the main chain amides on the “outer” β-strands of the PAD β-sheet make a series of hydrogen bonds with the template and primer strands. Additional DNA contacts are made by the thumb and the N-clasp, with the N-clasp effectively “locking” the thumb, fingers, palm domains and the PAD around the DNA (Fig. 1B).

### 8-oxoG(*syn*):A(*anti*) Hoogsteen base pair in the active site

**Figure 2:**
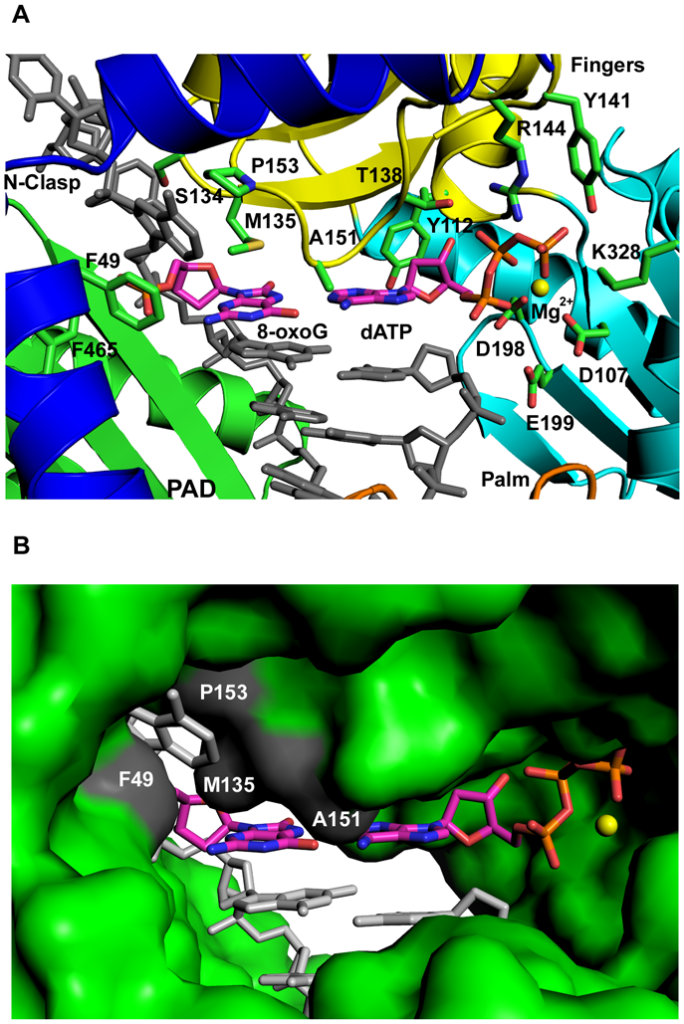
Hoogsteen base pairing between 8-oxoG(*syn*) and incoming dATP. (A) Close-up view of the Polκ active site cleft. Highlighted and labeled are the catalytic residues (D107, D198, and E199), residues apposed close to incoming dATP (Y112, T138, R144, Y141, and K328), 8-oxoG (M135 and A151), and the base 5*′* to 8-oxoG (F49 and P153). A putative Mg^2+^ ion is shown as a yellow sphere. (B) Molecular surface representation of the Polκ active site cleft. Highlighted in gray and labeled are residues apposed close to 8-oxoG and the base 5′ to it. A putative Mg^2+^ ion is shown as a yellow sphere.

**Figure 3:**
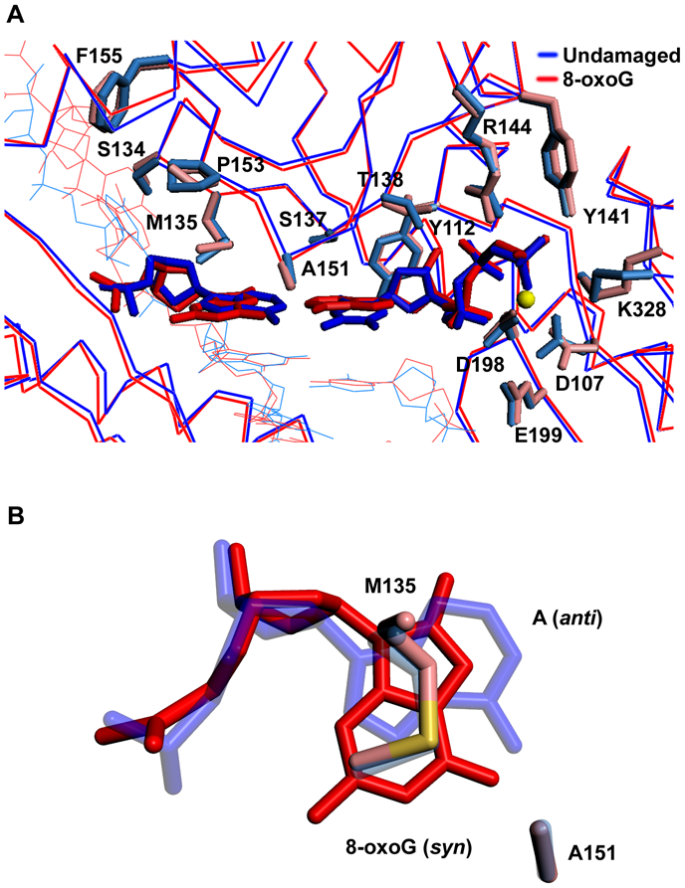
Damaged versus undamaged DNA. (A) Superimposition of the 8-oxoG complex (red) and the complex with undamaged DNA (blue; PDB code 2OH2). Highlighted are the template bases (8-oxoG and A), incoming dNTPs (dATP and dTTP) and residues within the active site cleft. A putative Mg^2+^ ion is shown as a yellow sphere. (B) Superposition of template 8-oxoG(*syn)* (red) and template A(*anti*) (blue). The figure shows the stacking of the M135 side chain over the template bases, and the relative position of A151.

The structure reveals an 8-oxoG(*syn*):A(*anti*) Hoogsteen base pair in the Polκ active site (Figs. 1 and 2). The template 8-oxoG lesion is rotated to the *syn* conformation, wherein its Hoogsteen edge (N7 and O^6^) is presented for hydrogen bonding with the Watson-Crick edge of dATP (N1 and N^6^), which remains in the *anti* conformation (Figs. 1C and 2). The C1′-C1′ distance across the 8-oxoG(*syn*):A(*anti*) Hoogsteen base pair is ∼10.96 Å, which is comparable to the distance (∼10.86 Å) in the nascent A(*anti*):T (*anti*) base pair in the ternary complex with undamaged DNA [24]. There is no major alteration in the polymerase structure, except for the slight reorientation of some residues in the vicinity of the lesion (described below) (Fig. 3A). The polymerase superimposes with an rms deviation of 0.54 Å when compared to the polymerase in the undamaged complex. The template-primer also binds in the same register as in the undamaged complex, and there is little or no movement of the N-clasp in accommodating an 8-oxoG adducted DNA. Incoming dATP binds with its triphosphate moiety interlaced between the fingers and palm domains, making hydrogen bonds with Tyr141 and Arg144 from the fingers domain and Lys328 from the palm domain (Fig. 2A). The catalytic residues, Asp107, Asp198 and Glu199, are clustered between the triphosphate moiety and the primer terminus (Fig. 2A). A Mg^2+^ ion occupies a position corresponding to “metal B” in replicative polymerases [29]–[31], and is coordinated in the basal octahedral plane by the unesterified oxygens of dATP β- and γ-phosphates and the carboxylates of Asp107 and Asp198, and at the apical positions by the α-phosphate and the main chain carbonyl of Met108. There is no density for a Mg^2+^ ion at a position analogous to “metal A” in replicative polymerases or in Y-family polymerases [13], [17]. However, as in the ternary complex with undamaged DNA, there is density for a water molecule, located ∼2 Å from the site normally occupied by metal A in replicative polymerases.

From the structure, the Polκ active site is well-adapted to accommodate an 8-oxoG lesion in the *syn* conformation for Hoogsteen base pairing with incoming dATP. The O8 of 8-oxoG (*syn*) is solvent exposed and does not sterically impinge on any residues in the Polκ active site (Fig. 2). The DNA template strand is also unaffected by the presence of 8-oxoG in the *syn* conformation. In addition, the *syn* conformation of 8-oxoG is stabilized by Met135 emanating from the fingers domain (Figs. 2 and 3). Compared to the undamaged complex, Met135 undergoes a slight change in conformation whereby the terminal atoms (Cγ-Cε) lie in a plane **∼**3.5****Å above 8-oxoG lesion and make van der Waals and stacking interactions (Figs. 2 and 3B). Thus, whereas in the undamaged complex Met135 lies primarily over the 5-membered ring of template A, in the 8-oxoG complex it covers almost the entire lesion (Fig. 3B). Supplementing Met135, Ala151 is in position to make van der Waals contacts with O6 of 8-oxoG(*syn*) (Figs. 2 and 3).

To examine whether Met135 contributes to the rotation of 8-oxoG into the *syn* conformation and thereby for Polκ's preference for A incorporation, we compared the catalytic efficiency of nucleotide incorporation opposite a non-damaged G and 8-oxoG by the wildtype Polκ and the mutant Polκ protein harboring a mutation of Met135 to alanine (M_135_A). As shown in Table 2, compared to wildtype, the Polκ M_135_A mutation resulted in a 36-fold decrease in C incorporation opposite non-damaged G. Thus, Met 135 plays an important role in the catalytic efficiency of Polκ, and the reduced activity of the mutant protein may derive from the involvement of Met 135 in stabilizing the nascent template base. Compared to its own catalytic efficiency for C incorporation opposite undamaged G, opposite 8-oxoG, M_135_A Polκ exhibited somewhat more of a decrease in C incorporation (56 fold) than the decrease observed for C incorporation opposite 8-oxoG by the wildtype protein (28 fold), whereas the incorporation of A opposite 8-oxoG was affected similarly for both the wildtype and mutant proteins (∼14 fold decrease). Thus, while Met135 has an important role in the catalytic efficiency of Polκ, possibly through stabilization of the template residue for proper base pairing with the incoming dNTP, it does not appear to contribute significantly to the rotation of 8-oxoG into the *syn* conformation.

**Table 2:** Kinetics of dCTP and dATP incorporation opposite undamaged G and 8-oxoG by Polκ.

Polκ (1–526)	Template: Incoming dNTP	k_cat_(min^−1^)	K_m_ (µM)	Efficiency k_cat_/K_m_	Fold decrease in efficiency
	G:C	6.8±0.5	5.1±1.7	1.3	-
Wild type	8-oxoG:C	1.1±0.07	23.9±6.1	4.6×10^−2^	28
	8-oxoG:A	1.0±0.03	11.1±1.4	9.0×10^−2^	14
	G:C	6.8±0.2	30.5±4.7	3.6×10^−2^	36 (1)^b^
M135A	8-oxoG:C	nd^a^	> 250	6.4×10^−4^	2040 (56)
	8-oxoG:A	10.5±1.1	71.6±7.5	2.8×10^−3^	455 (13)

a Since the amount of product formed remained linear throughout the dNTP concentrations, the efficiency was determined from the slope of the line.

b Numbers in parentheses indicate the fold reduction in nucleotide incorporation efficiency opposite 8-oxoG relative to nondamaged G for the M135A mutant Polκ protein.

## Discussion

**Figure 4:**
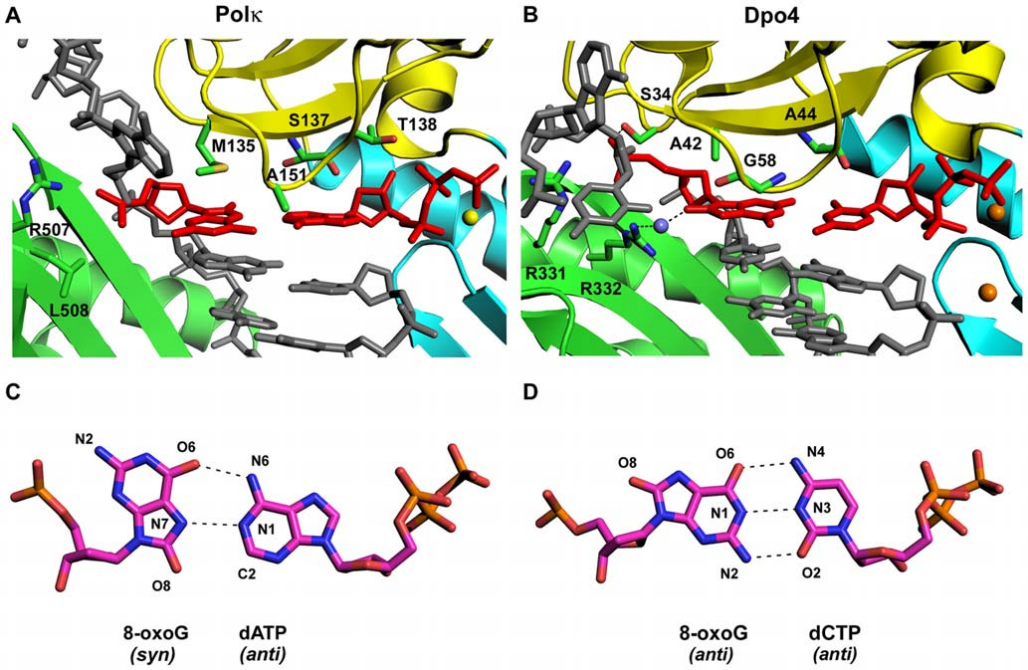
Comparison between Polκ and Dpo4 active site regions. Close up views of the Polκ (A) and Dpo4 (B) active site regions with template 8-oxoG. Highlighted and labeled are some of the residues implicated in stabilizing 8-oxoG (*syn*) in the Polκ active site region (M135 and A151) and 8-oxoG (*anti*) in the Dpo4 active site region (R331, R332 and S34). In the Polκ active site the putative Mg2^+^ ion is shown as a yellow sphere. In the Dpo4 complex [26], a water mediated hydrogen bond between R332 and O8 of 8-oxoG in Dpo4 is highlighted. The blue and orange spheres represent the water molecule and the calcium ions respectively. (C) A Hoogsteen 8-oxoG(*syn*):dATP (*anti*) base pair in the Polκ structure. (D) A W-C 8-oxoG(*anti*):dCTP (*anti*) base pair in the Dpo4 structure.

8-oxoG adducts are formed frequently by the attack of oxygen free radicals on DNA. They are amongst the most mutagenic lesions in cells because of their dual coding potential, where, in addition to normal base pairing of 8-oxoG in the *anti* conformation with dCTP [31], 8-oxoG in the *syn* conformation can base pair with dATP, causing G to T transversions. These misinsertion events are propagated due to the fact that 8-oxoG(*syn*):A base pairs do not register as mismatches during proofreading by replicative polymerases [32], [33]. DNA polymerases insert C or A opposite 8-oxoG at varying efficiencies, depending on the polymerase. For example, the replicative T7 and RB69 polymerases and the repair polymerase Polβ preferentially incorporate a C opposite 8-oxoG [32], [34], [35], while *Bacillus* Pol I preferentially incorporates an A [33]. Amongst Y-family polymerases, Polη and Dpo4 preferentially insert a C opposite 8-oxoG [24], [26], [27], whereas Polκ preferentially inserts an A opposite the lesion [36]. We show here that the Polκ active site is remarkably well-adapted to accommodate 8-oxoG in the *syn* conformation. That is, the polymerase and the template-primer are almost identical in their conformations to that in the undamaged DNA and present no steric hindrance to accommodating 8-oxoG in the *syn* conformation. In Polκ, the template base is contacted by Met135 emanating from the fingers domain. Met135 is unique to Polκ; the equivalent residue in other Y-family polymerases is typically smaller [22]. In Polη and Dpo4, for example, the equivalent residues are Ser58 and Ala42, respectively, which because of their smaller size would not be able to make the same number of van der Waals contacts to 8-oxoG(*syn*) as Met135 in Polκ. Mutation of Met135 in Polκ to alanine results in a 36 fold decrease in DNA synthetic activity, but seems not to significantly impact the rotation of 8-oxoG from *anti* to *syn*. Rather, the rotation of 8-oxoG to *syn* is likely a consequence of the steric clash between O8 of 8-oxoG and the template phosphate backbone. In the structure of Polκ with a template A in the active site [23], the distance of C8 of A to its 5′ phosphate of the backbone is ∼3.2–3.9****Å. Thus, maintaining the *anti* conformation of the template residue after substitution of an oxygen at the C8 position, as in 8-oxoG, would require a distortion of the DNA backbone. Such is the case for Dpo4, where structures with nascent 8-oxoG(*anti)*.C base pairs [26], [27] have revealed that the 5′ phosphate group of 8-oxoG flips by 180°, analogous to that observed with Polβ [34]. The phosphate group of 8-oxoG is stabilized in this position by hydrogen bonds with Arg331 and Arg332 from the PAD and Ser34 from the fingers domain (Fig. 4B), and additionally Arg332 forms a water mediated or a direct hydrogen bond to the O8 of 8-oxoG (*anti*) [26], [27]. Interestingly, this hydrogen bond is disrupted in the Dpo4 structure with a nascent 8-oxoG (*syn)*.A base pair [27], and it may partially account for Dpo4's preference for inserting dCTP opposite 8-oxoG. Intriguingly, neither Arg332 or Ser34 is present in Polκ. Ser34 in Dpo4 is on a segment that is not present in the Polκ fingers domain and Arg322 is substituted by a leucine (Leu508) (Fig. 4). The absence of these residues may shift the equilibrium of 8-oxoG from *anti* to *syn* in the Polκ active site. Polκ is also set apart from Dpo4 and other DNA polymerases by an N-clasp that interacts (via Phe49) with the phosphate and the nucleotide 5′ to 8-oxoG (Fig. 2). These interactions could hinder the rotation of the DNA backbone that relieves the steric overlap with O8 of 8-oxoG (*anti*) in Dpo4, and favor 8-oxoG(*syn)* for base-pairing with incoming dATP in Polκ. This is in contrast to T7 polymerase, where the reluctance to incorporate A opposite 8-oxoG is due to a lysine (Lys536) in the fingers domain that sterically and/or electrostatically clashes with the O8 of 8-oxoG in the *syn* conformation [37]. Indeed, the K536A mutant of T7 is the only replicative polymerase structure, to our knowledge, to show an 8-oxoG(*syn*):A base pair in the active site (at the insertion site) [37]. Taken together, the structures of Polκ and other DNA polymerases reveal unexpectedly high divergence - even amongst polymerases from the same family - in how they replicate an 8-oxoG DNA lesion.

## Methods

### Protein and DNA preparation

Polκ_19-526_, Polκ_1-526_ and Polκ_1-526_ M_135_A were purified from yeast strain BJ5464 harboring plasmids pBJ943, pBJ940 and pJRC10, respectfully as was described previously [22], [23]. The N-terminal fusion GST tags were removed by incubation with PreScission Protease (GE Healthcare) after an initial affinity chromatography step. For crystallization, the Pol κ_19-52_ protein was further purified by ion exchange (SP sepharose) and size exclusion (SD200) chromatography. The Polκ M_135_A mutation was generated by PCR using mutagenic oligonucleotides. The 13-nt primer for crystallization was synthesized with a dideoxycytosine at its 3′ end (GGGGGAAGGACTddC) and annealed to an 18-nt template synthesized with a 7,8-Dihydro-8-oxoguanosine at the 5^th^ position (CCTA(^.^8oxoG)^.^ GAGTCCTTCCCCC). Both oligonucleotides (W.M. Keck Facility-Yale University) were first purified by ion exchange chromatography (Q sepharose).

### Nucleotide incorporation assays

DNA synthesis assays were performed as described [38] using a 75mer oligonucleotide template containing a G or 8-oxoG residue at the 45^th^ position annealed to a 44mer oligonucleotide primer [25]. Reactions (5 µl) contained 25 mM Tris-HCl pH7.5, 5 mM MgCl_2_, 0.1 mg/ml BSA, 1 mM DTT, 10% glycerol, 10 nM DNA substrate and varying amounts of dCTP or dATP (0–500 µM). Assays contained 1 nM wildtype or mutant protein and were carried out for 5 min at 37°C. Reaction products were separated on 10% TBE-PAGE gels containing 8 M urea, and visualized by a phosphorimager (Molecular Dynamics). Kinetic parameters were determined by plotting the rate of product formation versus dNTP concentration and fit to the Michealis-Menten equation as described [38].

### Cocrystallization

Polκ at a final concentration of 0.5 mM was incubated with the 13-nt/18-nt primer/template in a buffer containing 25 mM HEPES (pH 7.0), 200 mM NaCl, 1 mM TCEP (Tris [2-carboxyethyl] phosphine hydrochloride), 10 mM MgCl_2_ and 10 mM dATP. Cocrystals were obtained from solutions containing 14% PEG 5000 monomethyl ether (w/v) (MME), 200 mM potassium acetate, 100 mM NaCl and 100 mM sodium cacodylate (pH 6.3). The cocrystals belong to the same space group (C222_1_) as the cocrystals reported previously with undamaged DNA [24], and have similar cell dimensions of a = 116.8 Å, b = 154.5 Å, and c = 217.3 Å. The cocrystals were cryoprotected for data collection by soaking them in mother liquor solutions containing increasing percentages of PEG 350 MME (5–30%), followed by flash-freezing in liquid nitrogen.

### Structure determination and refinement

X-ray data were recorded at Brookhaven National Laboratory (BNL, beamline X25). A native dataset to 3.2 Å was indexed, integrated and scaled using HKL2000 [39]. The dataset was then used to find a solution by molecular replacement (MR) using the polymerase from the Polκ ternary complex with undamaged DNA as a search model [24]. As expected, the program PHASER [40] found a unique MR solution with two protein molecules per asymmetric unit. Rigid body refinement with CNS [41] and subsequent electron density maps showed clear densities for the DNA, incoming dATP, and the 8-oxoG lesion. Iterative rounds of positional and B-factor refinement with CNS and model building with COOT [42] reduced the Rfree to 28.6%, with an Rcryst of 22.9%. The final model includes residues 25–224 and 281–518 for protein molecule A; residues 22–223 and 284–518 for protein molecule B; nucleotides 2–16 for template (T) and 3–13 for primer (P) bound to protein A, and nucleotides 4–17 for template (U) and 2–13 for primer (Q) bound to protein B; two incoming dATPs; 4 Mg^2+^; and 18 water molecules were also positioned in the density. Approximately 7% and 10% of the amino acids were built as alanines in molecule A and B (primarily at the N-terminus), respectively, due to the lack of density to accurately build the corresponding side chains.

### Structural analysis

The Polκ-8oxoG model has good stereochemistry, as shown by PROCHECK [43], with 84.4% of residues in the most favored regions of the Ramachandran plot and 2 outliers in the loop linking the thumb to the PAD domains. Figures were prepared using PyMol [44].
